# A comprehensive investigation of protein expression profiles in *L*. *monocytogenes* exposed to thermal abuse, mild acid, and salt stress conditions

**DOI:** 10.3389/fmicb.2023.1271787

**Published:** 2023-10-09

**Authors:** Federica D'Onofrio, Maria Schirone, Ivanka Krasteva, Manuela Tittarelli, Luigi Iannetti, Francesco Pomilio, Marina Torresi, Antonello Paparella, Nicola D'Alterio, Mirella Luciani

**Affiliations:** ^1^Department of Bioscience and Technology for Food, Agriculture and Environment, University of Teramo, Teramo, Italy; ^2^Istituto Zooprofilattico Sperimentale dell'Abruzzo e del Molise “G. Caporale”, Teramo, Italy

**Keywords:** *Listeria monocytogenes*, proteome, bioinformatic, STRING database, stressors, pathogen, virulence

## Abstract

Preventing *L. monocytogenes* infection is crucial for food safety, considering its widespread presence in the environment and its association with contaminated RTE foods. The pathogen's ability to persist under adverse conditions, for example, in food processing facilities, is linked to virulence and resistance mechanisms, including biofilm formation. In this study, the protein expression patterns of two *L. monocytogenes* 1/2a strains, grown under environmental stressors (mild acidic pH, thermal abuse, and high concentration of NaCl), were investigated. Protein identification and prediction were performed by nLC-ESI-MS/MS and nine different bioinformatic software programs, respectively. Gene enrichment analysis was carried out by STRING v11.05. A total of 1,215 proteins were identified, of which 335 were non-cytosolic proteins and 265 were immunogenic proteins. Proteomic analysis revealed differences in protein expression between *L. monocytogenes* strains in stressful conditions. The two strains exhibited unique protein expression profiles linked to stress response, virulence, and pathogenesis. Studying the proteomic profiles of such microorganisms provides information about adaptation and potential treatments, highlighting their genetic diversity and demonstrating the utility of bioinformatics and proteomics for a broader analysis of pathogens.

## 1. Introduction

*Listeria monocytogenes* is a pathogenic bacterium that is generally recognized as an important hazard in the food industry, as it causes outbreaks of listeriosis mainly in pregnant women, newborns, elderly, and individuals with compromised immune systems (Shevchuk, 2021). This microorganism is ubiquitous, and therefore the most effective control strategy for listeriosis involves the prevention of food contamination (Carpentier and Cerf, 2011; Dhama et al., 2015). The crucial niches for its transmission to animals, plants, and the food chain are mainly soil and water (Linke et al., 2014), and the outbreaks are often related to the consumption of contaminated ready-to-eat (RTE) foods, such as RTE meat products, soft cheeses, and fresh produce (Zhang et al., 2021). The pathogen is able to survive and persist under unfavorable conditions (i.e., desiccation, heat, high salt content, and refrigerated temperatures) in food processing facilities for extended periods of time due to ineffective cleaning and sanitation practices in difficult access sites, insufficient controls of movements of employees or equipment in areas with different risks of contamination, and a lack of design of preventive strategies (Rodríguez-López et al., 2018; Belias et al., 2022).

Foods are complex matrices in which bacteria can be exposed to natural stressful conditions with consequent physiological and structural changes in the cells, which allow them not only to withstand but also to grow and to express virulence-associated genes (Alvarez-Ordóñez et al., 2015; Fang et al., 2016). It is assumed that biofilm production by *L. monocytogenes* is one of the ways that permits increased resistance and persistence in the food chain (Lee et al., 2019), as cells resident in biofilm remain adhered to and protected by the extracellular polymeric matrix (Hobley et al., 2015). Furthermore, Li et al. (2020) demonstrated how some protein expressions, such as Lmo2193, encoded by the *oppD* gene, can affect positively both biofilm formation and virulence. However, the prevalence of *L. monocytogenes* in food facilities may not automatically be comparable to its occurrence in food products (Buchanan et al., 2017).

Therefore, rapid, accurate, sensitive, and high-throughput methods to detect and identify such microorganisms are important requirements for both the scientific community and the food industry (André et al., 2017). Traditionally, foodborne pathogen identification was carried out by culture-based approaches and the study of morphological, biochemical, and physiological microorganism characteristics. However, such techniques require some days or weeks before obtaining results, and dead cells or toxic substances cannot be revealed. Then, molecular techniques (i.e., DNA-based methods or antibody-based assays) have been developed to provide faster and more reliable data.

Currently, proteomics, genomics, metabolomics, and transcriptomics, denoted as “foodomics” when used together with bioinformatics tools, can give significant information on the biomolecules produced by food or organisms (Abril et al., 2022). These techniques have allowed scientific researchers to gain insights into the virulence mechanisms of *L. monocytogenes*, the genes and proteins involved in its pathogenesis, as well as the host–pathogen interactions that underlie listeriosis (Palumbo et al., 2022).

Proteomics enables the identification, characterization, and quantification of a comprehensive range of proteins upregulated or downregulated by a live organism when exposed to specific growth conditions (Chen et al., 2016), revealing protein function and expression (Böhme et al., 2013). Furthermore, proteomics methods are useful to identify structural and non-structural proteins involved in virulence as well as to investigate metabolic and physiological characteristics. Utilizing the combination of mass spectrometry analysis with different gel-based (i.e., SDS-PAGE, one- or two-dimensional gel electrophoresis) or shotgun proteomics approaches, researchers can detect bacterial infections more effectively, providing information on the proteins and the virulence factors involved in pathogenicity. In fact, understanding protein functions is crucial for the prevention and control of food pathogens, as well as for improving diagnosis (Zubair et al., 2022). Proteomic analyses of *L. monocytogenes* allow us to identify the proteins involved in the various aspects of its pathogenesis, including adhesion, invasion, intracellular survival, and virulence (Karthikeyan et al., 2019). Some studies have shown that *L. monocytogenes*-expressed proteins are implicated in the formation of cell surface structures to adhere to and invade host cells, but also in intracellular survival to change the host cell environment and promote bacterial growth (Birk et al., 2021).

In the present study, we investigated the protein expression patterns of *L. monocytogenes* involved in the adaptation to various stressful conditions using proteomic methods. Notably, previous studies have also explored similar approaches to gain insights into proteomic studies on the growth of such microorganisms and their adaptation to mild acid, thermal abuse, and high salt concentrations (D'Onofrio et al., 2023). For example, Mata et al. (2015) conducted a comprehensive proteomic analysis to examine the influence of strain genetics and temperature. Furthermore, Bowman et al. (2012) used similar techniques to study acid tolerance responses, while Abram et al. (2008) identified components contributing to acid and salt tolerance. However, proteomic studies on the growth and adaptation of *L. monocytogenes* to mild acid, low temperature, and high salt concentration at the same time are lacking. Moreover, profiling the whole protein expression to investigate systemic modifications in the microbial world is still a pioneering research field.

Therefore, the aim of the present study is the investigation of the protein expression patterns involved in the adaptation of two *L. monocytogenes* strains to various stressful conditions. A combination set of cardinal growth parameters (12°C as thermal abuse, maximum and minimum values of pH and NaCl), considered realistic for pork products, was carried out to observe the survival and multiplication of *L. monocytogenes* serotype 1/2a strains. Furthermore, this study aimed to identify the proteins involved in the virulent pathways during the exponential growth phase, comparing *in vitro* data with optimal growth conditions.

## 2. Materials and methods

### 2.1. Cultivation of *L. monocytogenes* serotype 1/2a strains

Two *L. monocytogenes* serotype 1/2a strains (268M “ATCC^®^ BAA-679™” as reference strain and 3178-CB-2018 as wild type, isolated from pork sausage) were grown at different conditions (C1-opt: 37°C, pH 7.0, NaCl 0.5%; C2: 37°C, pH 5.5, NaCl 7%; C3: 12°C, pH 7.0, NaCl 0.5%; C4: 12°C, pH 5.5, NaCl 7%). Biological and technical triplicates were performed for each experimental condition using Brain Heart Infusion (BHI) broth (Oxoid Thermo Fisher Scientific, Rodano, Italy) (NaCl 0.5%, pH 7.0) and modified BHI broth (NaCl 7%, pH 5.5), according to D'Onofrio et al. (2022). Bacterial cells were grown in a 15 mL tube (OD_600_ = 0.9), diluted 1:100 in BHI and modified BHI broth, and then cultivated under continuous agitation. Bacterial cells were collected at the late exponential growth phase (OD_600_ = 0.6), after 6 h, 3 days, 24 h, and 14 days for C1-opt, C2, C3, and C4, respectively and washed with sterile ice-cold 0.01 M phosphate-buffered saline (PBS) pH 7.0. The pellets were centrifuged without adding any cryoprotective agent (Eppendorf, Hamburg, Germany) at 5,600 × *g* at 4°C for 10 min and stored at −80°C until use.

### 2.2. Protein extraction

The proteins were extracted by CelLytic B Cell Lysis Reagent (Sigma-Aldrich, Milan, Italy) and CelLytic IB Inclusion Body Solubilization Reagent (Sigma-Aldrich, Milan, Italy). The lysates were precipitated by precipitation buffer (TCA 6.1 N), incubated for 45 min at 4°C, and centrifuged (10,000 *g* × 15 min at 4°C), washed with 100 μl of cold acetone, and dried under vacuum (Sanchez, 2001). The samples were resuspended in a solubilization solution (10 mM Tris–HCl; pH 7.5, 200 mM NaCl; 1 mM PMSF). The PierceTM BCA Protein Assay Kit (Thermo Fisher Scientific, Rodano, Italy) was used to evaluate protein concentrations. The protein extracts were resolved by SDS-PAGE loading 12 μg of proteins per well using NuPAGE^TM^ 4–12% Bis-Tris pre-cast gels (Life Technologies Thermo Fisher Scientific, Monza, Italy) at 200 V.

### 2.3. Mass spectrometry analysis

*L*. *monocytogenes* purified lysates were in-gel digested, excising the lanes with a scalpel (Shevchenko et al., 1996).

The gel lanes (Supplementary material) were subjected to a reduction-alkylation process by 10 mM dithiothreitol (DTT) and 55 mM iodoacetamide (IAA), respectively, and trypsin digested at 37°C overnight. Peptides were desalted by StageTip C18 (Rappsilber et al., 2003), dried, and resuspended in 5% formic acid. A peptide mixture (5 μL) was injected on a quadrupole Orbitrap Q-Exactive HF mass spectrometer coupled with a UHPLC Easy-nLC 1200 (Thermo Fisher Scientific, Waltham, MA, USA) with a 25 cm fused-silica emitter of 75 μm inner diameter (New Objective), packed in-house with ReproSil-Pur C18-AQ 1.9 μm beads (Dr. Maisch GmbH, Ammerbuch, Germany). Peptide separation was conducted with a single run time of 33 min using a linear gradient (23 min) from 95% solvent A (2% ACN and 0.1% formic acid) to 50% solvent B (80% acetonitrile and 0.1% formic acid) and from 50 to 100% solvent B (2 min) at a constant flow rate of 0.25 μl/min.

A data-dependent acquisition (DDA) top 15 method was performed. Survey full scan MS spectra (300–1,750 Th) were acquired in the Orbitrap with 60,000 resolution, AGC target 1e6, and IT 120 ms. For HCD spectra, resolution was set to 15,000, AGC target 1e5, IT 120 ms; normalized collision energy 28%, isolation width of 3.0 m/z, and a dynamic exclusion of 5 s.

### 2.4. MS data analysis

Raw MS files were processed using Proteome Discoverer (version 1.4.1.14, Thermo Fisher Scientific, Waltham, MA, USA). MS/MS peak lists were searched by Mascot engine (version 2.6.0, Matrix Science, Boston, MA, USA) against the database “uniprot_listeria_monocytogenes” [setting parameters: enzyme trypsin; maximum missed cleavage 2; fixed modification carbamidomethylation (C); variable modification oxidation (M); and protein N-terminal acetylation; peptide mass tolerance 10 ppm; MS/MS tolerance 20 mmu]. Scaffold (version Scaffold_4.3.3, Proteome Software Inc., Portland, OR) was used to validate peptide and protein identification by the Scaffold Local FDR algorithm (peptide and protein threshold >95.0 and 99.0%, respectively). Furthermore, protein identifications were accepted if they contained at least two identified peptides. Protein probabilities were assigned by the Protein Prophet algorithm (Nesvizhskii et al., 2003). The proteins were grouped to satisfy the principles of parsimony, and those sharing significant peptide evidence were grouped into clusters. The proteins identified in at least two out of three biological replicates were included in the protein prediction analysis. The unique and commonly identified proteins among experimental conditions were grouped by Venn Diagram using Venny 2.0 software.

### 2.5. Protein prediction analysis

The proteins were analyzed by five software programs to evaluate subcellular localization (SCL): PSORTb version 3.0.2 and CELLO version 2.5 were used as bacterial protein SCL predictors (Krogh et al., 2001; Nesvizhskii et al., 2003; Rahman et al., 2008). LipoP 1.0 server was used to predict lipoproteins and to differentiate lipoprotein signal peptides (SPs) (Shevchenko et al., 1996); TMHMM Server version 2.0 was used to evaluate the prediction of transmembrane helices (Rappsilber et al., 2003; Perez-Riverol et al., 2022), and SignalP 4.1 server predicts the presence and location of SP cleavage sites in amino acid sequences (Nesvizhskii et al., 2003). The immunogenic proteins were examined to evaluate the immunogenic candidates by means of VirulentPred and VaxiJen 2.0 servers (Garp and Gupta, 2008; Petersen et al., 2009; Jespersen et al., 2017). VirulentPred is predictive software for the identification of bacterial virulent proteins. This method is founded on a two-layer cascade support vector machine (SVM) approach, employing a preset threshold of 0.0. VaxiJen operates as an autonomous server focused on foretelling the physicochemical traits of antigens and proteins without relying on sequence alignment. In terms of data evaluation through VaxiJen, the selection of proteins is influenced by their “Minimum Adhesin Probability” (MAP) score. In VaxiJen, a MAP varying between 0.4 and 0.5 indicates that a protein is a potential adhesin or possesses adhesin-like characteristics (D'Onofrio et al., 2022). The immunogenic proteins were analyzed to predict the protein immunogenic regions by NetSurfP version 1.1 (Srivastava et al., 2021) and BepiPred version 1.0 server, the former to predict the secondary structure and the relative surface exposure of the individual amino acid residues, and the latter to predict the B-cell epitopes (Lin et al., 2009).

### 2.6. STRING v.11.05 analysis

The *in silico* enrichment analysis was performed using STRING (Search Tool for the Retrieval of Interacting Genes/Proteins) software version 11.05. The proteins with epitopes exposed to solvent (EETS), identified among each condition, were listed in the Supplementary material. The proteins were analyzed, setting a minimum required interaction score of 0.700, and visualized by the network. The proteins were clustered by the *K*-means clustering algorithm to highlight the different enriched pathways. Different colors were used to evaluate the functional characteristics of clustered proteins. Gene Ontology Biological Processes (GOBPs), Gene Ontology Molecular Functions (GOMFs), and text mining functions were used to describe the network functional enrichment analysis. Moreover, GOBPs were obtained from ShinyGO 0.77 for 3178-CB-2018 C2 and C3 conditions. Data visualization was performed using R version 4.3.0, utilizing the ggplot2 package.

## 3. Results

In the present study, the protein expression profiles of *L. monocytogenes* in response to different environmental conditions, considered realistic for pork products, and specifically mild acidic pH, thermal abuse, and high osmolarity level (C1-opt: 37°C, pH 7.0, NaCl 0.5%; C2: 37°C, pH 5.5, NaCl 7%; C3: 12°C, pH 7.0, NaCl 0.5%; C4: 12°C, pH 5.5, NaCl 7%), were examined.

*L. monocytogenes* serotype 1/2a 268M (accession number: ASM19603v1; https://www.ncbi.nlm.nih.gov/datasets/genome/GCF_000196035.1/) was chosen because its sequence was already analyzed in a previous study (Palma et al., 2022), and it is the reference strain used in many publications on the genomic organization of this species. *L. monocytogenes* serotype 1/2a 3178-CB-2018 (BioSample accession SAMN37347693, BioProject: PRJNA1015181) was studied for its genetic characteristics, which are similar to a strain that caused an outbreak in Italy (Orsini et al., 2018).

The primary focus was to discern the proteins implicated in the virulence pathways of *L. monocytogenes* during its exponential growth phase and compare them with those expressed under optimal growth conditions (C1-opt). Through the integration of proteomic and immunoinformatic results, it was possible to identify mechanisms enabling *L. monocytogene*s to endure stressful environments that could potentially enhance its pathogenicity.

**Figure 1:**
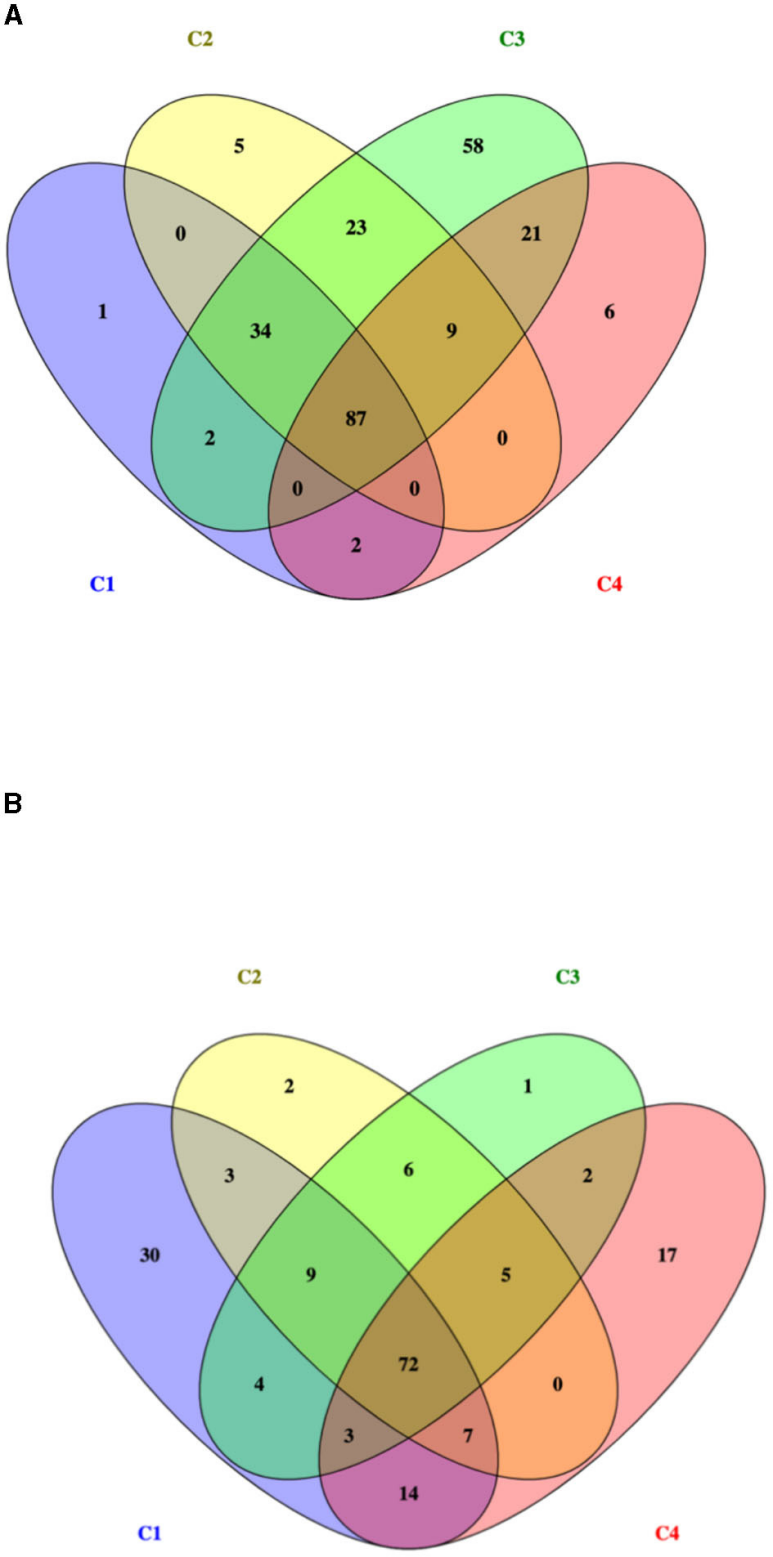
Venn diagram of identified proteins for two *L. monocytogenes* serotype 1/2a strains: **(A)** 268M and **(B)** 3178-CB-2018. C1-opt: 37°C, pH 7.0, NaCl 0.5%; C2: 37°C, pH 5.5, NaCl 7%; C3: 12°C, pH 7.0, NaCl 0.5%; and C4: 12°C, pH 5.5, NaCl 7%.

**Figure 2:**
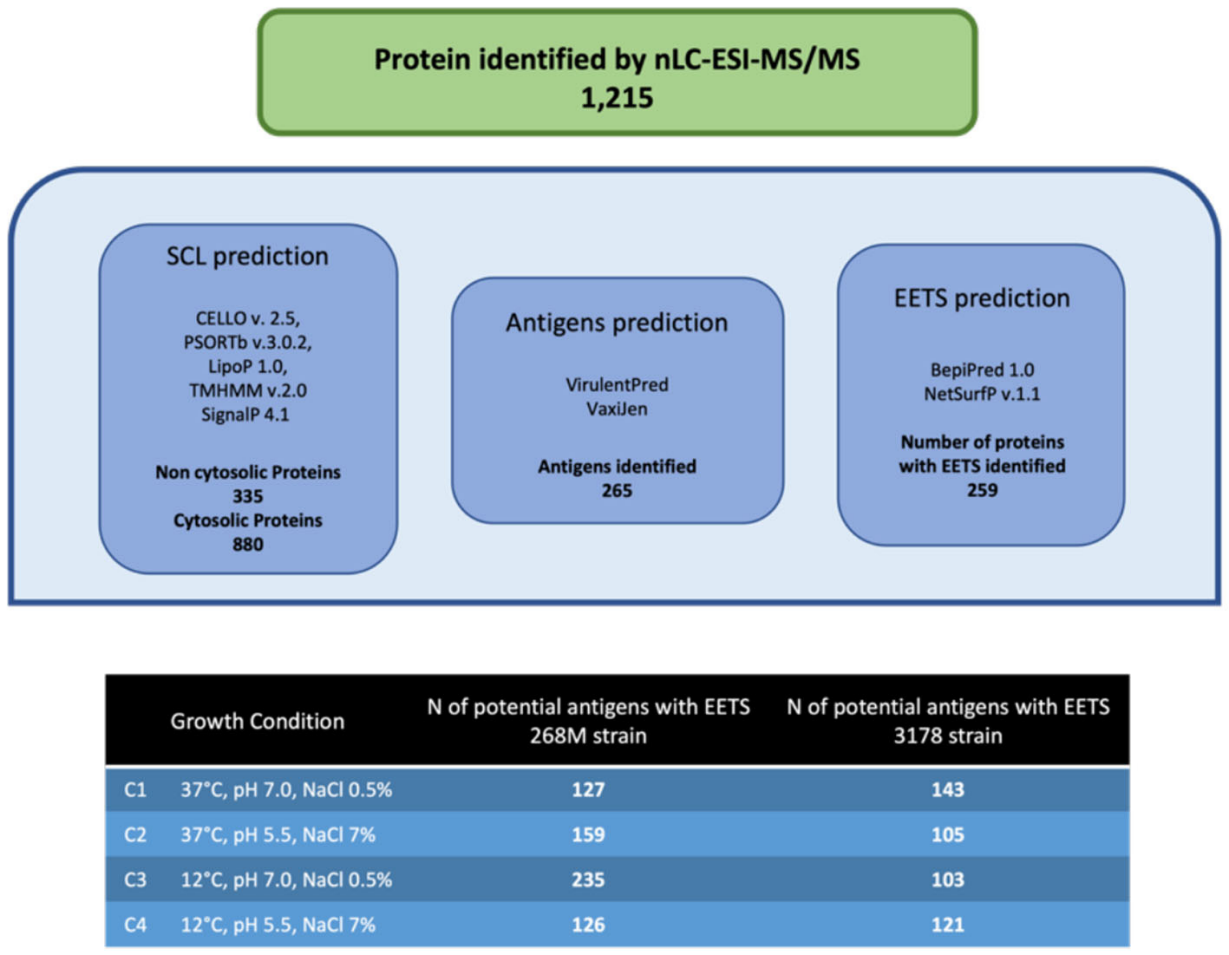
Immunoinformatic workflow for immunogenic protein identification of 268M and 3178-CB-2018 *L. monocytogenes* strains after exposure to C1-opt: 37°C, pH 7.0, NaCl 0.5%; C2: 37°C, pH 5.5, NaCl 7%; C3: 12°C, pH 7.0, NaCl 0.5%; and C4: 12°C, pH 5.5, NaCl 7%.

The total number of proteins identified by nLC-ESI-MS/MS was 1,215, considering all experimental conditions. Unique and overlapping proteins for each cultivated strain (268M and 3178-CB-2018) were grouped by Venn diagram (Figure 1). The reference (268M) and the wild-type (3178-CB-2018) strains presented 126 vs. 142 proteins in C1-opt, 158 vs. 104 in C2, 234 vs. 102 in C3, and 125 vs. 120 in C4, respectively. Out of these proteins, 87 and 72 were common in 268M and 3178-CB-2018, respectively. The SCL was performed by five software programs (CELLO version 2.5, PSORTb version 3.0.2, LipoP 1.0 Server, TMHMM Server version 2.0, and SignalP 4.1 Server). The SCL analysis led to the identification of 335 non-cytosolic proteins (NCPs). The immunogenic proteins predicted among NCP by VirulentPred and VaxiJen 2.0 servers were 265. The candidates with EETS predicted via NetSurfP v.1.1 and BepiPred v.1.0 were 259 (Figure 2). All results are available as Supplementary material.

**Figure 3:**
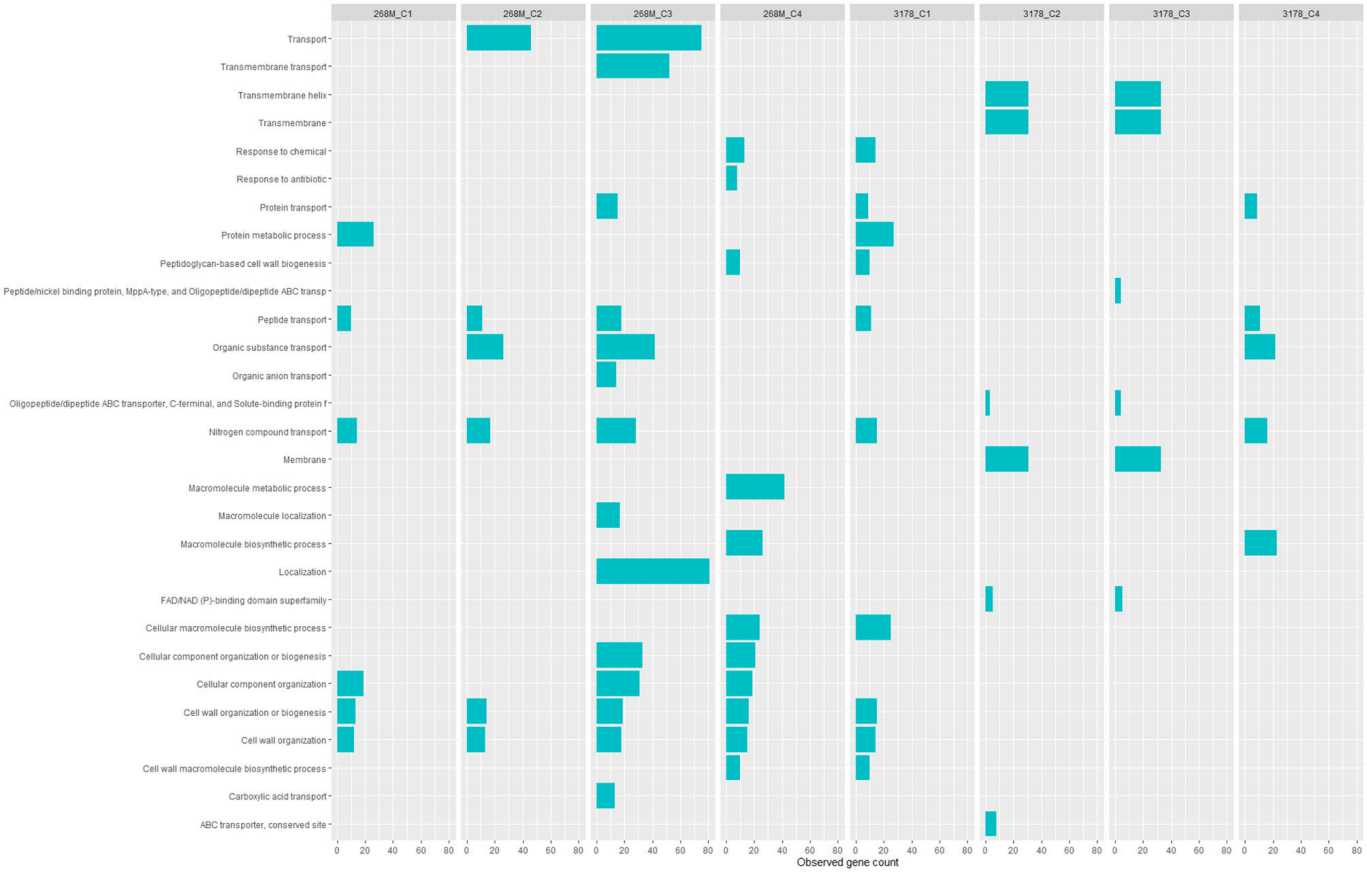
Data visualization obtained by R version 4.3.0 (ggplot2 package) of *in silico* enrichment analysis performed by ShinyGO 0.77, of GOBPs expressed by the 268M and 3178-CB-2018 *L. monocytogenes* strains after exposure to C1-opt: 37°C, pH 7.0, NaCl 0.5%; C2: 37°C, pH 5.5, NaCl 7%; C3: 12°C, pH 7.0, NaCl 0.5%; and C4: 12°C, pH 5.5, NaCl 7%. The blue bars indicate the number of genes (*x*-axes) that are codified for the proteins involved in each biological process.

**Figure 4:**
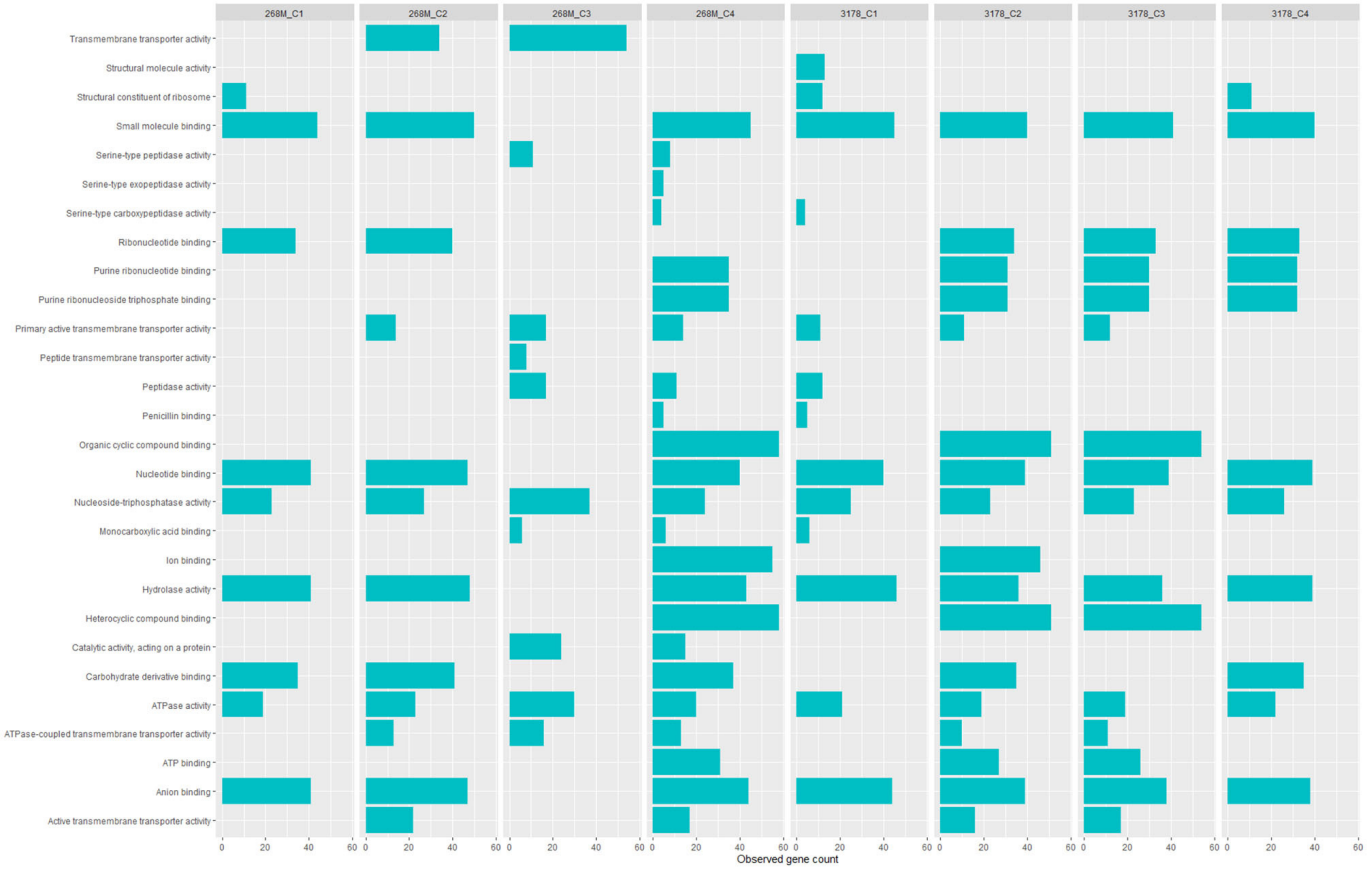
Data visualization obtained by R version 4.3.0 (ggplot2 package) of *in silico* enrichment analysis performed by ShinyGO 0.77 of GOMFs expressed by the 268M and 3178-CB-2018 *L. monocytogenes* strains after exposure to C1-opt: 37°C, pH 7.0, NaCl 0.5%; C2: 37°C, pH 5.5, NaCl 7%; C3: 12°C, pH 7.0, NaCl 0.5%; and C4: 12°C, pH 5.5, NaCl 7%. The blue bars indicate the number of genes (*x*-axes) that are codified for the proteins involved in each biological process.

GOBPs and GOMFs enriched for each experimental condition are shown in Figures 3, 4. GOBPs and GOMFs represent the biological processes in which genes are involved and the molecular functions of genes (i.e., enzyme activity, receptor binding, or transporter activity), respectively. All the proteins identified among C1-opt, C2, C3, and C4 for both strains were used to perform the enrichment analysis. The most enriched GOBPs in C1-opt, C2, C3, and C4 were protein metabolic process (26 genes), transport (46 genes), localization (81 genes), and macromolecule metabolic process (42 genes) for the 268M strain, whereas protein metabolic process (27 genes), membrane (31 genes), membrane (33 genes), and macromolecule biosynthetic process (23 genes) were the most enriched GOBPs for 3178-CB-2018. On the other side, the most enriched GOMFs for the 268M strain in C1-opt and C2 were 44 and 50 genes in small molecule binding, respectively, while in C3 and C4, they were transmembrane transporter activity (54 genes) and organic and heterocyclic compound binding (58 genes), respectively. GOMFs results for the wild-type strain showed that hydrolase activity (46 genes), organic and heterocyclic compound binding (51 genes), organic and heterocyclic compound binding (54 genes), and small molecule binding (40 genes) were enriched for C1-opt, C2, C3, and C4.

The *in silico* enrichment analysis led to visualize eight different networks based on the experimental growth conditions for 268M and 3178-CB-2018. The proteins are represented by nodes and identified by their code genes. The edges represent the protein–protein interactions and the different interactions among them. The nodes of the same colors are related to the same molecular pathway.

**Figure 5:**
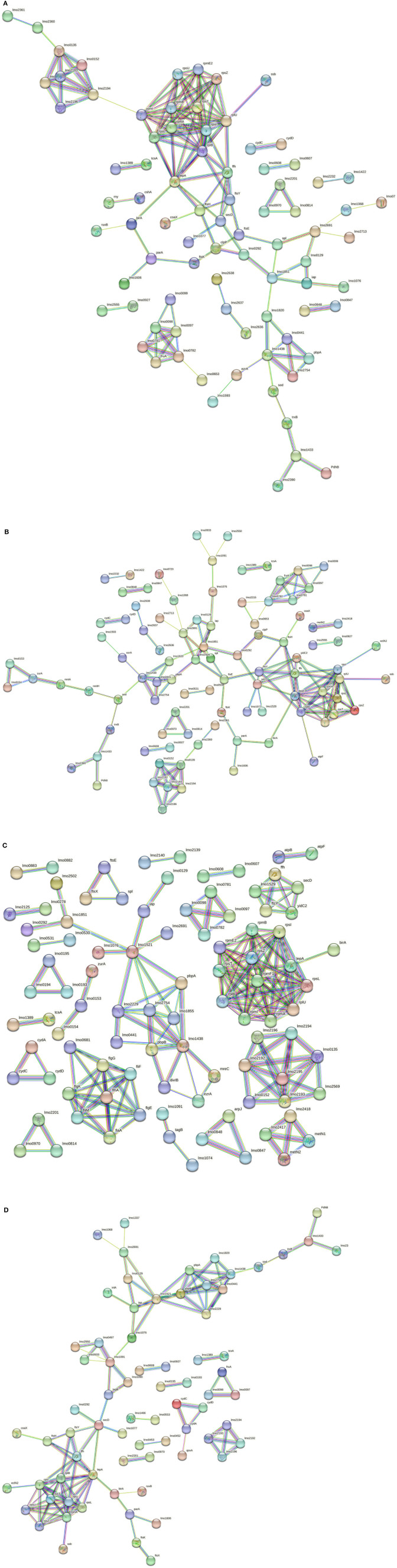
**(A)** Network STRING v11.05 diagram showing the expressed proteins by the 268M *L. monocytogenes* 1/2a strain after exposure to C1-opt: 37°C, pH 7.0, NaCl 0.5%. **(B)** Network STRING v11.05 diagram showing the expressed proteins by the 268M *L. monocytogenes* 1/2a strain after exposure to C2: 37°C, pH 5.5, NaCl 7%. **(C)** Network STRING v11.05 diagram showing the expressed proteins by 268 M *L. monocytogenes* 1/2a strain after exposure to C3: 12°C, pH 7.0, NaCl 0.5%. **(D)** Network STRING v11.05 diagram showing the expressed proteins by 268 M *L. monocytogenes* 1/2a strain after exposure to C4: 12°C, pH 5.5, NaCl 7%. Nodes = protein; edges = protein–protein interactions; known interactions = pink and light blue colors; predicted interactions = green, red, and blue colors; other interactions = yellow, black, and gray colors; empty nodes = proteins of unknown 3D structure; filled nodes = some 3D structure is known or predicted.

In 268M, 126 nodes and 338 edges for C1-opt (Figure 5A) were displayed. A total of 94 proteins were identified and annotated in 6 GOBPs, while 289 proteins were identified and included in 9 GOMFs. A total of 158 nodes and 152 edges were identified in response to high osmolarity level and low pH (C2), as shown in Figure 5B. A total of 127 and 406 proteins were identified and annotated across 6 GOBPs and 12 GOMFs, respectively. The analysis of the effects of thermal abuse (C3) identified 234 nodes and 314 edges (Figure 5C). In addition, a total of 456 proteins were annotated across 14 GOBPs, while 406 proteins were explained across 10 GOMFs. Against thermal abuse, mild acid conditions, and high osmolarity levels, 125 nodes and 124 edges were identified (Figure 5D). Furthermore, the analysis identified a total of 204 proteins annotated across 11 GOBPs, along with 623 proteins noted across 23 GOMFs.

**Figure 6:**
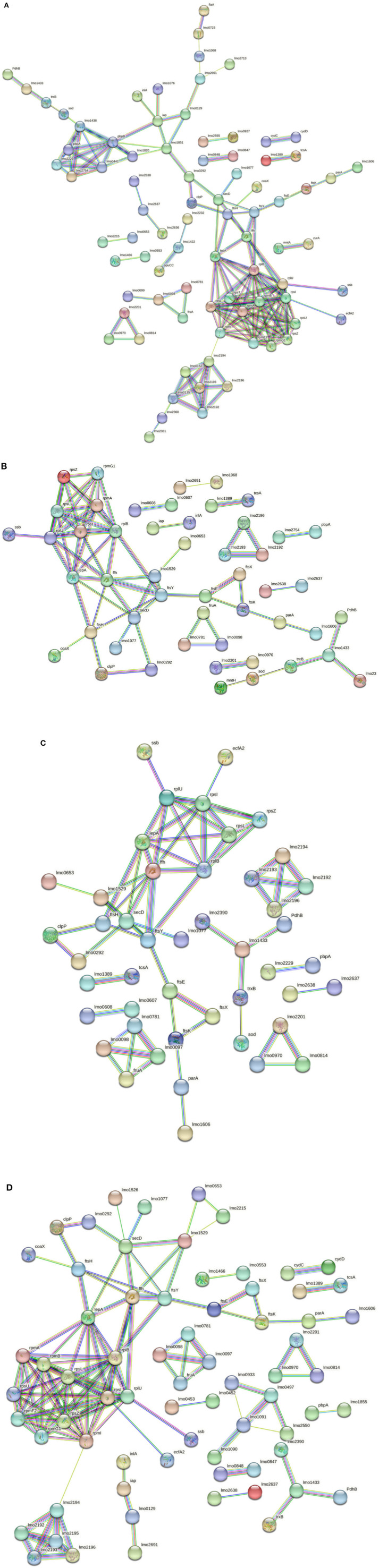
**(A)** Network STRING v11.05 diagram showing the expressed proteins by the 3178-CB-2018 *L. monocytogenes* 1/2a strain after exposure to C1-opt: 37°C, pH 7.0, NaCl 0.5%. **(B)** Network STRING v11.05 diagram showing the expressed proteins by the 3178-CB-2018 *L. monocytogenes* 1/2a strain after exposure to C2: 37°C, pH 5.5, NaCl 7%. **(C)** Network STRING v11.05 diagram showing the expressed proteins by the 3178-CB-2018 *L. monocytogenes* 1/2a strain after exposure to C3: 12°C, pH 7.0, NaCl 0.5%. **(D)** Network STRING v11.05 diagram showing the expressed proteins by the 3178-CB-2018 *L. monocytogenes* 1/2a strain after exposure to C4: 12°C, pH 5.5, NaCl 7%. Nodes = protein; edges = protein–protein interactions; known interactions = pink and light blue colors; predicted interactions = green, red, and blue colors; other interactions = yellow, black, and gray colors; empty nodes = proteins of unknown 3D structure; filled nodes = some 3D structure is known or predicted.

Considering the 3178-CB-2018 strain, 142 nodes and 167 edges were identified for C1-opt (Figure 6A). The gene enrichment analysis highlighted 10 GOBPs and 13 GOMFs, including 150 and 284 proteins, respectively. To face the high osmolarity level combined with the low pH (C2), 6 GOBFs and 17 GOMFs were enriched among a network of 104 nodes and 72 edges (Figure 6B). The analysis also identified 109 and 503 proteins annotated across GOBPs and GOMFs, respectively.

Thermal abuse (C3) analysis revealed a network containing 102 nodes and 65 edges (Figure 6C). Furthermore, the analysis identified 112 and 463 proteins that were annotated in 6 GOBPs and 15 GOMFs, respectively. Considering C4, 120 nodes and 133 edges were identified (Figure 6D). Furthermore, 5 GOBFs and 11 GOMFs were significantly enriched by 81 and 347 proteins, respectively.

## 4. Discussion

Several enriched BPs were identified across the different growth conditions in both *L. monocytogenes* 1/2a strains. In C1-opt, 268M and 3178-CB-2018 exhibited enrichment in the BPs related to protein metabolic processes and cellular component organization. Such shared enrichment suggests that the strains focused on maintaining cellular homeostasis rather than survival under stress conditions, and this feature was common in both strains. In C2, 268M prominently enriched the BPs associated with transport, particularly organic substance transport, cell wall organization, and biogenesis. In contrast, 3178-CB-2018 showed significant enrichment in the pathways related to membrane, transmembrane, and transmembrane helix. This may indicate that both *L. monocytogenes* strains (268M and 3178-CB-2018) modified cell wall and membrane structures, probably as an adaptive strategy to maintain cellular homeostasis and resist the acidic pH of the C2 condition (Cheng et al., 2015). To face thermal abuse, both strains activated genes related to the synthesis and localization of flagella. Furthermore, to withstand lower temperatures, the strains modified the cellular structures, not only at the level of the cell wall but also inside the membrane, both below 25°C. Under the most stressful conditions, the investigated strains prioritized cell wall organization and biogenesis of BPs as a strategy to improve resistance to cold temperatures and defend cellular components. Additionally, peptidoglycan-based cell wall biogenesis, associated with penicillin-binding proteins (Pbp), plays a significant role in these adaptations (Bucur et al., 2018). Notably, both strains extended their modifications beyond the cell wall, also affecting membrane structures (Guariglia-Oropeza et al., 2018; Quereda et al., 2021). Although the *L. monocytogenes* strains used in this study belonged to the same serogroup (1/2a), it needs to be pointed out that they are different clonal complexes (CCs): in fact, 268M and 3178-CB-2018 are CC9 and CC7, respectively. This phylogenetic distance may have led to distinct protein expression profiles. Among all the experimental conditions, the same clusters were identified for both investigated strains.

The oligopeptide permease (Opp) complex and the ribosomal protein cluster (RPC) are essential for the survival and growth of bacterial cells. The Opp complex and the RPC are involved in the uptake of nutrients and in the fundamental process of protein synthesis, respectively (Calamita et al., 2018). The former is constituted by OppA (*lmo2196*), OppC (*lmo2194*), OppD (*lmo2193*), and OppF (*lmo2192*): they are peptide transporter subunits implicated in virulence gene expression as part of cell-to-cell signaling regulation. In fact, OppD seems to be involved in biofilm formation and virulence pathways. Moreover, the Opp complex includes Lmo2360 and Lmo2361, which are transmembrane permeases as in the 268M strain (Disson et al., 2021). The RPC is related to translation and is conserved across bacterial species (*rplU, rpmB, rpsT, rpmI, rpsU, rpsZ, rpmE2, rplB, rpsI, rpsL*, and *rpmA*) (Hain et al., 2008). These ribosomal proteins play a critical role in ensuring accurate and efficient translation of mRNA into proteins. Mutations in RPC proteins have been shown to affect ribosome biogenesis and function, leading to a variety of cellular defects and diseases (Calamita et al., 2018). The locus of enterocyte effacement proliferation A *(*LepA) and signal recognition particle (SRP) protein Ffh (*ffh)* are involved in signaling pathways to induce molecular switches during stressor exposure. LepA is required to face stress conditions such as nutrient deprivation, heat shock, and oxidative stress. Ffh binds to the signal peptide of the secreted protein, ensuring proper insertion and folding. Ffh has also been implicated in the regulation of cell division (Pleitner et al., 2014). The energy coupling factors A2 (EcfA2) and ATP synthase F (AtpF) are part of the cluster in the presence of a low pH and high osmolarity (C2); these proteins are connected to RPC. EcfA2 clusters with RPC also in C4; it is a subunit transporter of ThiT, which is an integral membrane complex required for thiamine recognition and transport. *L. monocytogenes* needs thiamine as a cofactor because its genome lacks a partial list of genes involved in thiamine biosynthesis (Madeo et al., 2012). Moreover, ThiT is necessary to enhance acid tolerance and intracellular growth during pathogenesis (Schauer et al., 2009). Overall, the presence of EcfA2 and AtpF in this cluster suggests that they are involved in the energy metabolism and protein synthesis required for the survival and adaptation of *L. monocytogenes* under different environmental conditions. AtpF is a subunit of plasma membrane proton-transporting ATP synthase complex coupling factor (F) 0, which is responsible for generating ATP by using the proton motive force across the bacterial membrane and contributes to facing acidity stress in *L. monocytogenes* by maintaining membrane potential and pH homeostasis (Bucur et al., 2018). In C3, AtpF was connected to AtpB, which seems to be overexpressed in response to various stress conditions, such as low pH combined with anaerobiosis, and in this study was expressed to face thermal abuse (Chakravarty et al., 2021).

In C2, the connection between LepA and YidC2 suggests a potential role for LepA in protein folding and insertion during nutrient transport (Burg-Golani et al., 2013). The fact that YidC2 was identified in response to thermal abuse and was clustered in the translocase complex further highlights its importance in the adaptation of *L. monocytogenes* to different environmental conditions. ParA and FtsK, which were present in all investigated conditions, are implicated in the optimal replication of *L. monocytogenes* in macrophages, as these proteins are important for cell division and the control of protein complex localization (Fischer et al., 2022). In the same cluster, genes involved in DNA metabolism and transport, such as *lmo1606*, DNA repair *ruvB*, and transcriptional regulator *birA*, are included. The presence of the first two genes listed above in the same cluster as *lepA* and *yidC2* in C2 suggests that these proteins could also play a role in DNA maintenance and repair during nutrient transport and adaptation to different environmental conditions. Then, the transcriptional regulator *birA* in this cluster may also be involved in the regulation of gene expression related to nutrient transport and stress response (Burg-Golani et al., 2013; Fischer et al., 2022).

In all experimental conditions, one cluster related to sugar uptake was present. Lmo0097, Lmo0098, Lmo0781, and Lmo0782 are kinase proteins involved in the carbohydrate phosphotransferase system (PTS) and are related to fructose and mannose-specific PTS. Moreover, they are positively regulated by the stress response factor σ*B*, which also contributes to host entry by *L. monocytogenes* (Mattila et al., 2020). The fructose permease IIC component (FruA) protein is connected to this cluster and is positively regulated by the *agr* gene. Such genes have a main role in the *quorum sensing* system, which is associated with cell survival, adhesion, biofilm formation, invasion, and the infectivity of host cells (Pinheiro et al., 2018).

Under the optimal growth condition (C1-opt), surface proteins that contribute to *L. monocytogenes* host cell adhesion clustered together. Lmo1068 is involved in attachment and biofilm formation (Tiong and Muriana, 2016), Lmo0723 in chemotaxis, and Lmo2713 is upregulated by the σ*B* factor during host cell invasion (Neuhaus et al., 2013; Piercey et al., 2016). These proteins are also visualized in cluster 3 (C3), while Iap protein was identified in all studied conditions along with autolysin protein (Lmo1076), muramidase protein MurA (*lmo2691*) (Tiong and Muriana, 2016), and Lmo0129, which is involved in the host gastrointestinal tract colonization (Desvaux et al., 2010).

Chaperones and proteases contribute to virulence and stress tolerance. Caseinolytic protein-P (ClpP) is a subunit of the ATP-dependent Clp protease complex, which is involved in the adaptive response of *L. monocytogenes* during the infection process and upregulates listeriolysin O (LLO) production at 37°C. Moreover, a similar study on a different Gram-positive pathogen, *Staphylococcus aureus*, showed that it is required for motility and growth at high temperatures (Savijoki et al., 2020). Lmo0292 protein, also known as “high temperature requirement A” (HtrA), acts as a chaperone and serine protease (Abfalter et al., 2019). ClpP and Lmo0292 were present in all conditions. In such clusters, proteinases of the Fts family (FtsY, FtsH, FtsE, and FtsK) and other proteinases (Lmo1851 and Lmo1077) are also included, and they are involved in cell wall metabolism and in wall teichoic acid (WTA) biosynthesis and modification, respectively. Those proteins seem to be upregulated in the presence of stress factors, such as carnocyclin A (CCLA), which is an antimicrobial peptide used against *L. monocytogenes* in RTE meat products (Camejo et al., 2009; Liu et al., 2014).

The penicillin-binding protein (Pbp) family is involved in cell wall metabolism, antimicrobial resistance, and infections. Lmo0441, PbpA (*lmo1855*), Pbp5 (*lmo2754*), and Pbp3 (*lmo1438*) were present in all experimental conditions, except for PbpB (*lmo2039*) that was expressed only in C3 and C4 of 268M strain, indicating a different cell wall structure when exposed to various stressors. As regards the 3178-CB-2018 strain, the above-cited Pbp were all expressed at the optimal condition (C1-opt), while Lmo2754, Lmo2229, and Lmo1855 were identified in C2, C3, and C4, respectively (Shin et al., 2010; Lima et al., 2011; Fischer et al., 2020). Guinae et al. (2006) demonstrated that strains lacking Pbp3 and Pbp5 exhibit attenuated virulence. The upregulation of some of these proteins in response to stressors, such as antimicrobial peptides, suggests that *L. monocytogenes* has evolved mechanisms to resist host defense systems, and this may contribute to its ability to cause infection and illnesses. The importance of Pbp3 and Pbp5 in virulence further highlights the role of cell wall metabolism in the pathogenicity of the microorganism.

The CBS domain is found in both prokaryotes and eukaryotes, with an important role in protein–protein interactions for processes, such as cell adhesion, cell activation, and molecular recognition (Oliver et al., 2010). The CBS domain (Lmo2232) was present in C1-opt both for 268M clustered to Lmo1422 and for 3178-CB-2018 to Lmo1422 and OpuCC. Lmo1422 and OpuCC are subunits of an ABC transporter complex: the former is involved in glycine betaine transport, protection by oxidative damage, and the SOS response of *L. monocytogenes* (Zhang et al., 2022), and the latter is concerned with the carnitine accumulation pathway (Van Der Veen et al., 2010). Moreover, both proteins are part of the σ*B*-dependent osmotic stress resistance systems (Liu et al., 2019). These differences suggest that 3178-CB-2018 in C1-opt has a distinct surface protein profile that may contribute to its ability to adhere to both surfaces and host cells.

Proteins associated with the oxidative stress response were expressed in different conditions, suggesting *L. monocytogenes* adaptation to oxidative environments. In C1-opt for both 268M and 3178-CB-2018, superoxide dismutase (SOD) was connected to Pbp3 in the network. This protein is classified as an ROS scavenger and, together with both thioredoxin reductase (TrxB) and glutathione reductase (Lmo1433); it aims to address oxidative stress in bacterial cells (Savijoki et al., 2020). Hain et al. (2008) determined that Lmo1433 is σ*B*-dependent in *L. monocytogenes*. PdhB is a stress response protein involved in the repair of DNA during oxidative stress (D'Onofrio et al., 2022). The same cluster was found in C4 for both strains. In C2 for 268M, more proteins related to oxidative stress were identified, as the MntA protein is linked to SOD by MntH; these proteins are expressed in this experimental condition to cope with low pH and high osmolarity (Zeng et al., 2019). In fact, manganese (Mn) transporters (MntH and MntB) were found to be highly expressed after exposure to mild acid stress (pH 5) (Wu et al., 2023).

ZurA, Lmo0153, and Lmo0154, involved as complexes in zinc uptake systems (ZurAM and ZinABC), were both expressed in C2 and C3 of 268M.

Only in C3 of 268M, there was a cluster formed by four thermosensors (Lmo2417, Lmo2418, Lmo2419, and MetN1), upregulated by the *prfA* gene. These proteins appear to be upregulated during the exponential growth phase and when the pathogen invades the host intestinal lumen. Among the ABC transporter systems regulated to cope with low temperatures, the Lmo0607–Lmo0608 complex, identified in all experimental conditions of 268M, acts as a multidrug transport system (Krawczyk-Balska et al., 2021). Arpj is an ABC transporter subunit for arginine, expressed only in C3 during intracellular replication and acid shock, while Lmo0847–Lmo0848 complex subunits act as glutamine ABC transporters and were found in all conditions (Neuhaus et al., 2013). Lmo0193–Lmo0195 complex acts as an ABC transporter and is upregulated during osmotic stress (C4) (Bergholz et al., 2012).

Lmo1388 (*tcsA*)–Lmo1389 are two-component system (TCS) proteins, positively regulated by σ*B* regulon (Oliver et al., 2010) and present in all conditions for both strains.

Lmo0530 is part of a five-gene operon (*lmo0527–lmo0531*), which seems to be σ^*B*^and σ^*A*^ dependent. It is involved in the enhanced synthesis of exopolysaccharides in response to osmolarity changes, facilitating bacterial survival under this stress condition (Ribeiro et al., 2014). In fact, Lmo0530 was expressed not only at 12°C but also in C2 and C3 of the 268M strain. Chen et al. (2014) and Köseoglu et al. (2015) demonstrated the involvement of cyclic diguanylate (c-di-GMP) signaling in *L. monocytogenes* and its impact on various bacterial behaviors, including the production of an exopolysaccharide, tolerance to stressors, and virulence.

To cope with low temperatures, *L. monocytogenes* synthesizes type II branched-chain fatty acids. Lmo0970 (FabI) is the regulatory protein reductase of type II in the fatty acid synthesis of *L. monocytogenes* and is essential together with Lmo2201 (FabF*-*B-ketoacyl-acyl-carrier-protein-synthase complex) and Lmo0814 (FabK) (Yao et al., 2016). In fact, this cluster was observed in C3 to face thermal abuse and also in C2 and C4, to overcome high salt content and low pH.

*TagB, lmo1074*, and *lmo1091* are specific genes of the *L. monocytogenes* serovar 1/2a strain: the first two encode for cell wall-associated proteins (tail-anchored translocation permeases), while the second is for a glycosyl transferase that is involved in cell wall biosynthesis. Being strain-specific, these proteins could have a definite impact on host–pathogen interaction (Chatterjee et al., 2006) and were present in C3 and C4 for 268M and only in C4 for 3178-CB-2018.

Genes involved in flagellar biosynthesis, such as *flhA*, were only observed in C3, in response to thermal abuse. In particular, induced by *lmo0681, flaA* codes for flagellin, which is the principal component of the bacterial flagellum and is usually expressed below 25°C and under specific stress conditions, such as the presence of detergents (Way et al., 2004; Hain et al., 2012). FliM is a complex subunit of the flagellar motor switch, essential for the transmembrane transport of protons and rotation of the flagella, thus enabling the bacterial cell to become motile with the help of FliF proteins, which are involved in flagellar assembly (Bigot et al., 2005). Flagellar structural genes are also part of the cluster, such as *flgK* (hook-associated protein), *flgE* (flagellar hook protein), and *flgG* (basal body rod proteins).

Lmo0553 and Lmo1466 proteins are part of the cyclic di-adenosine monophosphate (c-di-AMP) complex, which is a largely conserved second messenger essential for *L. monocytogenes* growth, stress responses, antibiotic resistance, cellular morphology, and virulence (Witte et al., 2013). C-di-AMP acts on the intracellular signaling pathway and is an important component of innate immune detection during infection (Sureka et al., 2014). Witte et al. (2013) highlighted the importance of c-di-AMP as a critical signaling molecule for *L. monocytogenes*, essential for bacterial replication, cell wall stability, and pathogenicity.

In C1-opt, 3178-CB-2018 expressed cell surface proteins that contribute to both surface and host cell adhesion. Specifically, in contrast to C1-opt for 268M, 3178-CB-2018 expressed InlA and FlaA, which are linked to the proteins Iap and Lmo0723, respectively.

On the other hand, there were several proteins present in 268M under the optimal condition that were absent in 3178-CB-2018. These include EzrA involved in cell division, Lmo1593 in iron–sulfur cofactor synthesis, RuvB and BirA in DNA metabolism and transcription, respectively, and Lmo0653, which is a hypothetical protein that is likely elaborated in lipoteichoic acid metabolism and could contribute to nisin resistance in *L. monocytogenes*, according to Pang et al. (2022).

To face high osmolarity and low pH (C2), the 3178-CB-2018 *L. monocytogenes* strain appears to produce fewer proteins than those expressed by the strain 268M grown under the same conditions. Despite this, InlA was only found in the wild-type strain and not in the reference strain, and therefore the presence of InlA in 3178-CB-2018 suggests that it may have a greater ability to infect host cells than 268M under high osmolarity and low pH conditions.

The same response was observed in the presence of thermal abuse (C3). However, several proteins clustered only in 3178-CB-2018, including LepA involved in signaling pathways, the fts proteinases family, Lmo1077 involved in wall teichoic acid (WTA) biosynthesis and modification, ClpP and ParA related to cell division, PdhB, Lmo1606 implicated in DNA metabolism, and Lmo0653. Moreover, thermal abuse induced the expression of several proteins that appear to be involved in *L. monocytogenes* pathogenesis, such as Lmo1526 (Knudsen et al., 2012) and Lmo2215; the latter is expressed by the *lmo2215* gene, which is one of the genes most associated with survival and stress response of *L. monocytogenes*, especially in meat and meat products (Tanui et al., 2022).

Lmo1529 is a YajC translocase preprotein subunit associated with protein secretion and codified by genes important for *L. monocytogenes* virulence (Muchaamba et al., 2022).

The *lmo2637* gene encodes for an EET-linked lipoprotein, PplA, while the *lmo2638* gene encodes for an EET-linked NADH dehydrogenase involved in ethanolamine (EA) metabolism (Zeng et al., 2021). EA is a source of carbon and nitrogen derived from the eukaryotic cell membrane; it can be used by a specific microbial redox metabolic subsystem called the bacterial microcompartment (BMC), inducing the activation of EA utilization (*eut*) genes, which are also linked to *L. monocytogenes* pathogenesis and growth (Zeng et al., 2021).

## 5. Conclusion

Investigating the proteomic profiles of *L. monocytogenes* under different stress conditions is of great importance for several reasons. In fact, understanding the molecular mechanisms that allow the pathogen to adapt and survive under stressful conditions is crucial for the development of effective strategies to control and prevent its spread. This study provides valuable insights into the expression patterns of specific genes and proteins under different stress conditions, which can be used to identify potential targets to discriminate between hypovirulent and hypervirulent strains of *L. monocytogenes*.

Furthermore, the study highlights the differences between the proteome profiles of two strains of *L. monocytogenes* grown under exposure to different stressors. This information can be used to better understand the genetic and phenotypic diversity of such a pathogen and to identify factors that contribute to its virulence and pathogenicity.

Finally, our results demonstrate the power of bioinformatic and proteomic approaches to analyze large datasets and identify important patterns and associations that would be difficult to detect using traditional laboratory methods. This methodology can be applied to other pathogens and stress conditions to gain a better understanding of their biology and pathogenesis.

## Data availability statement

The original contributions presented in the study are included in the article/Supplementary material, further inquiries can be directed to the corresponding author.

## Author contributions

FD'O: Methodology, Writing—original draft, Data curation, Formal analysis, Writing—review and editing. MS: Conceptualization, Supervision, Writing—original draft, Writing—review and editing. IK: Data curation, Formal analysis, and Methodology. MTi: Investigation and Visualization. LI: Investigation, Resources, Supervision, Writing—review and editing. FP: Funding acquisition, Investigation, and Visualization. MTo: Supervision, Writing—review and editing. AP: Supervision, Writing—review and editing. ND'A: Investigation, Visualization and Funding. ML: Conceptualization, Supervision, Writing—original draft, Writing—review and editing.
