# Supplementary material for: A comprehensive investigation of protein expression profiles in L. monocytogenes exposed to thermal abuse, mild acid, and salt stress conditions
Source: Front Microbiol. 2023 Oct 9;14:1271787. doi: 10.3389/fmicb.2023.1271787 (PMC10591339; doi:10.3389/fmicb.2023.1271787)
Supplement: Supplementary file 5 [file Image_1.pdf]

A comprehensive investigation of protein expression profiles in *L. monocytogenes* exposed to thermal abuse, mild acid, and salt stress conditions

Federica D'Onofrio[1,2], Maria Schirone[1*], Ivanka Krasteva[2], Manuela Tittarelli[2], Luigi Iannetti[2], Francesco Pomilio[2], Marina Torresi[2], Antonello Paparella[1], Nicola D'Alterio[2] and Mirella Luciani[2]

[1]Department of Bioscience and Technology for Food, Agriculture and Environment, University of Teramo, Teramo, Italy

[2]Istituto Zooprofilattico Sperimentale dell'Abruzzo e del Molise "G. Caporale", Teramo, Italy

*** Correspondence:**

Maria Schirone

mschirone@unite.it

Supplementary Figures

1.

St C1 C2 C3 C4

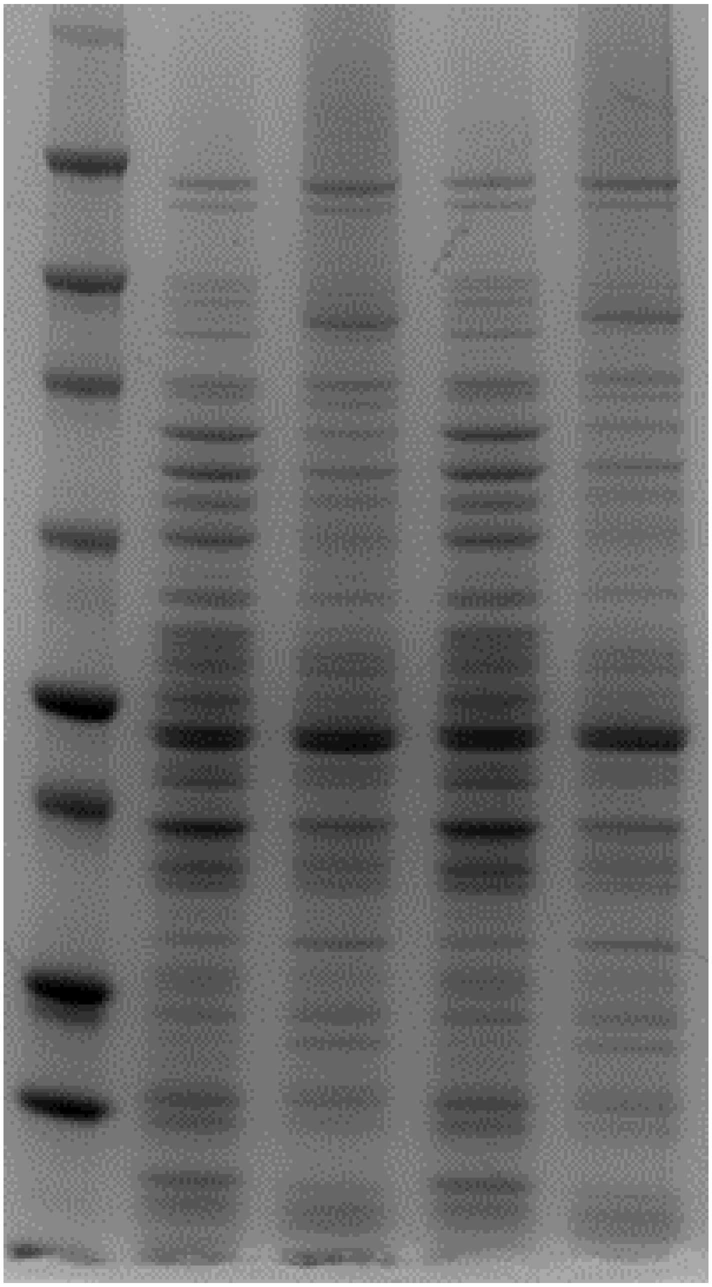

Supplementary Figure 1. SDS PAGE Coomassie stain: Protein banding patterns of *L. monocytogenes* 268M, cultivated at four different environmental conditions. St = Standard; C1 = 37◦C, pH 7.0, NaCl 0.5%; C2 = 37◦C, pH 5.5, NaCl 7%; C3 = 12◦C, pH 7.0, NaCl 0.5%; and C4 = 12◦C, pH 5.5, NaCl 7%.

St C1 C2 C3 C4

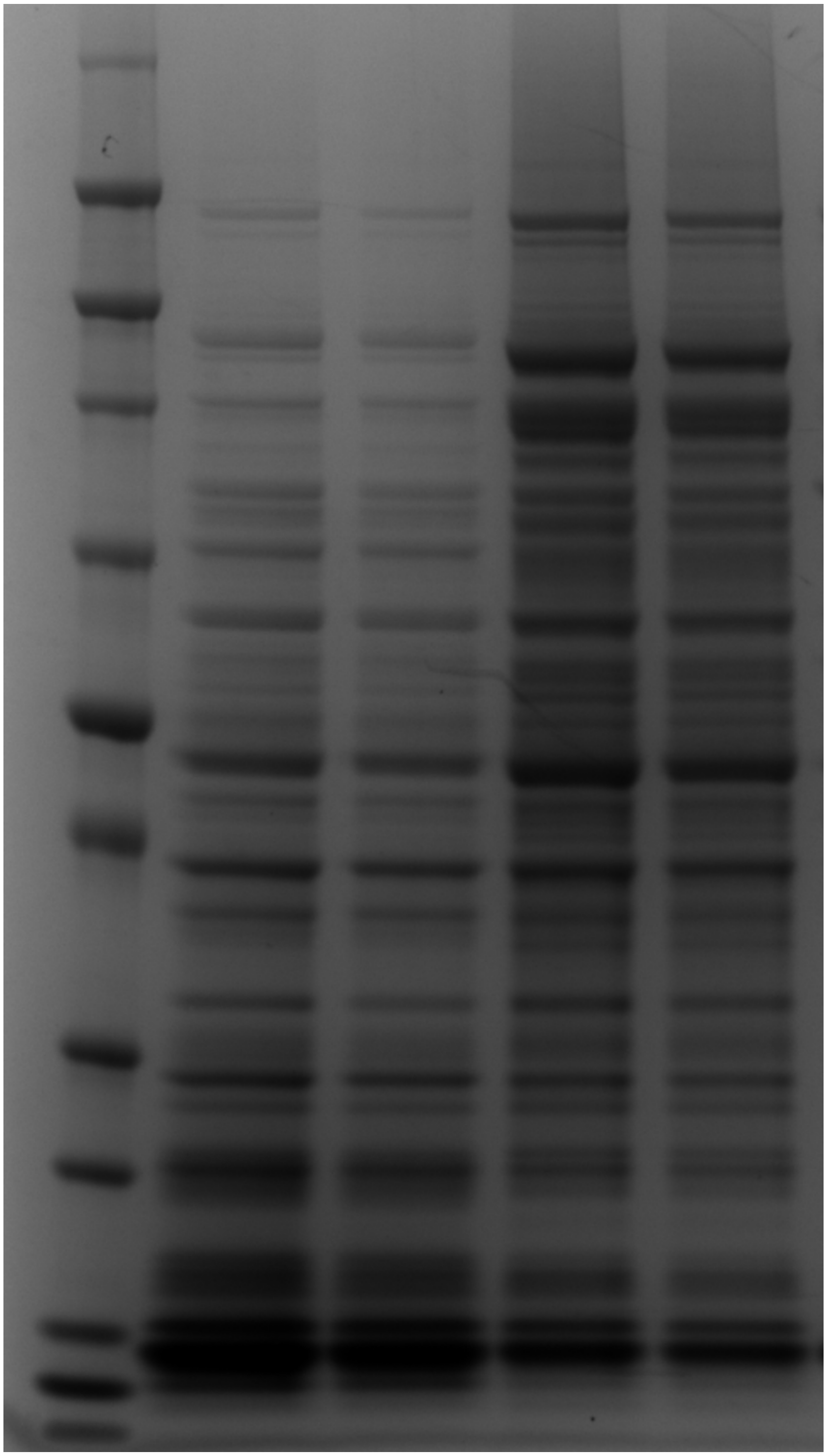

Supplementary Figure 2. SDS PAGE Coomassie stain: Protein banding patterns of *L. monocytogenes* 3178, cultivated at four different environmental conditions. St = Standard; C1 = 37◦C, pH 7.0, NaCl 0.5%; C2 = 37◦C, pH 5.5, NaCl 7%; C3 = 12◦C, pH 7.0, NaCl 0.5%; and C4 = 12◦C, pH 5.5, NaCl 7%.
